# Hida Scan in The Follow-Up of Biliary-Enteric Anastomoses

**DOI:** 10.1155/1988/89673

**Published:** 1988

**Authors:** G. Belli, G. Romano, A. Monaco, M. L. Santangelo

**Affiliations:** 1 Department of General Surgery and Organs Transplantation University of Naples Naples Italy; 2 via Cimarosa 2/A Naples 80127 Italy

## Abstract

In order to assess the patency and function of biliary-enteric anastomoses performed in our Department of Surgery, 21 patients entered the following study, provided an informed consent was obtained. All the patients were affected by benign biliary tract diseases and underwent either Roux-en-Y hepaticojejunostomy (11 cases), or side-to-side choledochoduodenostomy (10 cases). The 21 patients were evaluated with Tc-99m-HIDA scanning at intervals of 20 days–36 months after the surgical procedure (mean 14 months). The images were obtained after intravenous injection of the radioactive medium (5 mCi) and the scans were taken at 1 min (1 frame/s), 3 min (1 frame/10 s), and 56 min (1 frame/2 min). The data were analyzed by a Digital PDP 11/34 Computer System. This method allowed us to assess each individual patient for the patency of the anastomosis and, by computer analysis, to build up a profile of the timing of the passage of the radioactive medium through the anastomosis; a delayed passage across the anastomosis was always pathological.

In conclusion, the 99m-Tc-HIDA scanning used in our study for long-term follow-up of biliary-enteric anastomoses is reliable and allows an assessment of prognosis.

## INTRODUCTION

Hepaticojejunostomy (HJ) and choledochoduodenostomy (CD) represent the most commonly used biliary enteric anastomoses for cholelithiasis and bile duct reconstructive surgery.[1] Complications are relatively common; leakage and anastomotic breakdown usually present early on, whereas stenosis and cholangitis occur months or years after surgery[2–4]. The incidence of postoperative stenosis has been reported as 0.8–23%[4].

There is considerable variability in the criteria chosen to assess the long-term results of biliary by-pass operations[2,5,6]: the follow-up usually reported is too short (< 2 years) and, even when adequate follow-up is provided, an objective method of assessing the long-term patency and function of biliary anastomosis is lacking.

Ultrasonography is not always feasible and is frequently unreliable[7]. Percutaneous transhepatic cholangiography (PTC) is not justified for follow-up studies in asymptomatic patients. Cholescintigraphy with Tc-99m-labeled analogue of iminoadiacetic acid (HIDA), introduced for the diagnosis of suspected acute cholecystitis, is now being successfully applied in the evaluation of the postoperative

* Reprint requests and correspondence to: Giulio Belli, MD, via Cimarosa 2/A, 80127 Naples, Italy.

patient[7]. This study was undertaken to evaluate the role and limits of cholescintigraphy in 21 patients on whom biliary enteric anastomosis was performed.

## MATERIALS AND METHODS

In order to assess the patency and the function of biliary-enteric anastomosis, 21 patients entered the study. All the patients were affected by benign biliary tract diseases. Eleven patients had had Roux-en-Y HJ (one with associated pancreatico-jejunostomy for chronic pancreatitis). Ten patients had side-to-side CD. Besides having the usual laboratory tests, particularly serum alkaline phosphatase, all the patients were evaluated with Tc-99m-HIDA scanning at variable intervals after the surgical procedure (range 20 days–36 months, mean 14 months).

Images were obtained after intravenous injection of the radioactive medium (5m Ci). Scans were taken at 1 min (1 frame/s), 3 min (1 frame/10 s), and 56 min (1 frame/2 min). The data were analyzed using a digital PDP 11/34 computer system. After initial evaluation of the blood pool phase, hepatic function was studied by building up a profile of hepatocellular uptake, whereas assessment of anastomotic patency and function was obtained by visualization of ductual time activity dynamics (biliary-to-bowel transit less or more than 1 hour and pre- and postanastomotic dilatation). The results were considered good or bad according to HIDA findings, and compared to the clinical course, laboratory tests, sonography in all patients, PTC in one patient and endoscopic retrograde cholangiography (ERC) in another.

## RESULTS

The results are summarized in Table 1. Eighteen patients (10 HJ and eight CD), all considered to have a good result, showed intestinal excretion in less than one hour. Normal laboratory tests and a favorable clinical course (follow-up: 6–24 months) were observed for all these patients; sonography showed postoperative duct dilatation in three out of eight patients shown to have this by the HIDA finding. The most likely explanation for bile duct dilatation without delayed intestinal excretion is

**Table 1**

| *Results* | *Number* | | *Anastomosis outcome* |
|---|---|---|---|
| Intestinal excretion in one hour without ductal dilatation | 15 | 9 HJ | Good |
| | | 6 CD | Good |
| Intestinal excretion in one hour with ductal dilatation | 3 | 1 HJ | Good |
| | | 2 CD | Good |
| Intestinal excretion in more than one hour without ductal dilatation | 2 | 1 HJ[a] | Bad |
| | | 1 CD | Doubt (false positive) |
| Intestinal excretion in more than one hour with ductal dilatation | 1 | CD[b] | Bad |

[a] Stenosis confirmed by PTC.
[b] Stenosis and multiple stones confirmed by ERC.

long-standing preoperative stasis resulting in bile duct dilatation that may persist after the operation without affecting the anastomotic function.

Three patients showed intestinal excretion in more than one hour on the HIDA scan and were considered to have a poor result. Of these, one patient (HJ) developed recurrent cholangitis three months after the operation in spite of normal postoperative laboratory tests and sonographic findings; the suspicion of progressive anastomotic stricture assessed at early postoperative HIDA scanning was confirmed by PTC. This patient underwent a successful transhepatic balloon dilatation of the stricture, confirmed by the subsequent clinical course and HIDA scans performed six months and two years later.

A second patient (CD), considered to have a bad result, showed intestinal excretion in more than one hour and ductal dilatation on the HIDA scan one month after the operation. Bilirubin serum levels were normal, while a doubled value of alkaline phosphatase was observed. Sonography confirmed ductal dilation but did not reveal the cause. In spite of an asymptomatic clinical course and normal serum bilirubin levels, ERC was performed four months after the operation because of persistent high levels of alkaline phosphatase and delayed intestinal excretion on repeated cholescintigraphy. ERC showed stricture of the anastomosis with sludge and multiple stones in the biliary tract. A Roux-en-Y HJ was later successfully performed on this patient.

The third patient (CD) with a poor result was known to have an underlying hepatic parenchymal disease and did not show ductal dilatation as confirmed by sonography. Based on normal laboratory tests, negative sonographic findings and an asymptomatic clinical course, this patient was followed up for three years and considered a false positive result of the HIDA scan.

## DISCUSSION

In the follow-up of patients on whom biliary-enteric anastomosis is performed, there is need for a non-invasive diagnostic method that allows appropriate visualization of the anastomosis and objective evaluation of its patency and function. Frequently, the patient's history, physical examination and biochemical tests are inconclusive[8,9].

Presence of air or reflux of barium into the biliary tree demonstrated during an upper gastro-intestinal series is not conclusive evidence of good anastomotic function in patients with choledochoduodenostomy. These are indirect signs by a non-physiologic procedure that require retrograde flow of contrast[7]

Endoscopic retrograde cholangiography can be performed only in patients who have had anastomoses to the duodenum. This is an invasive technique with a possible risk of cholangitis and is best not used as a follow-up method.

Sonography has definite limitations: presence of air in the intestinal loop and the biliary tree results in loss of sensitivity in about 50% of cases[10]; it does not allow correct assessment of anastomosis function. PTC provides an excellent visualization of the anastomotic complex but is invasive, difficult to perform in non-jaundiced patients and cannot, therefore, be accepted as first-level examination at follow-up.

Tc-99m-HIDA cholescintigraphy is the only non-invasive procedure available that can accurately assess the function of biliary-enteric anastomosis in an antegrade "physiologic" manner regardless of the level of the by-pass[7].

In our small series, cholescintigraphy correctly visualized the anastomosis in all the patients. It has been our experience, as well as that of others, that, when intestinal activity is identified in the first 60 min after injection of Tc-99m-HIDA, patency of the by-pass is confirmed[7,11,12], regardless of evidence of ductal dilatation. The latter can be consequent on long-standing pre-operative obstruction and may not be a significant pathological finding.

Whenever delayed intestinal excretion (more than one hour) is associated with ductal dilatation, stenosis is usually present, as was the case in the patient with stricture of the choledochoduodenostomy.

The significance of delayed intestinal excretion associated with normal bile ducts is controversial. In a study by Weissmann, such a finding was always pathological[7]. In the two patients of our series with this feature, anastomotic stenosis could be confirmed only in one; the other patient known to have an underlying hepatic parenchymal disease was followed for three years, and good function of the anastomosis was confirmed by his subsequent asymptomatic clinical course, normal laboratory tests and negative sonography findings. It is likely that hepatocellular compromise could be responsible for delayed intestinal excretion in such patients. This case should be considered a false positive result.

The major weakness of cholescintigraphy is its limited anatomic resolution and inability to determine the nature of obstruction (stones, tumor relapse etc.). Nevertheless, cholescintigraphy remains the best non-invasive method for routine assessment of patency or obstruction of a biliary-enteric anastomosis[7,11,12].

The prognostic significance of cholescintigraphy requires further study but it may be valuable in this respect, as suggested in our patient with delayed intestinal excretion on early postoperative HIDA scan but with negative findings on laboratory tests and sonography. Whenever stenosis is suspected, PTC or ERC should be performed so as to define properly the anatomic pattern and to allow appropriate planning of re-operation.

## INVITED COMMENTARY

The authors report that Tc-99m-HIDA scanning was useful in long-term follow-up of 21 patients with biliary-enteric anastomoses. They demonstrated a "good" result by scintigraphy in 18 patients, a "bad" result in two, and one "false positive." These results suggest a 95% accuracy for this non-invasive test. The authors state that scintigraphy may demonstrate abnormal function when biochemical tests of liver function and ultrasonic findings are normal. They also point out that, in comparison to endoscopic retrograde of percutaneous transhepatic cholangiography, HIDA scanning provides functional data and is safer and more comfortable. These advantages for scintigraphic evaluation of biliary-enteric anastomes·also apply in comparisons with computerized tomography, magnetic resonance imaging, and percutaneous liver biopsy.

In this study from the University of Naples, scans were taken at 1, 3 and 56 min after injection of the radionuclide. The two parameters evaluated were ductal dilatation and intestinal uptake at 1. Ductal dilatation, however, did not correlate very well with clinical outcome. Other parameters that were not studied were intestinal appearance time and hepatic uptake and clearance time and rate. Moreover, these latter data can be individually calculated for the right and left hepatic lobes. To obtain these additional data, scans need to be taken at more points in time during the first hour after injection, and counting has to be performed over specific areas of interest.

Theoretically, lobar hepatic uptake and clearance rates and intestinal appearance time would provide more data on biliary-enteric anastomotic function than the single parameter of gut uptake at 1. These additional data might be especially useful for following-up patients over time and in patients with hepatic parenchymal disease. Moreover, a significant percentage of patients who require biliary-enteric anastomoses have some degree of secondary biliary fibrosis or cirrhosis. In these patients, serial studies may also be helpful in assessment of both liver and anastomotic function over time.

Other ongoing issues in the management of patients with biliary-enteric anastomoses are the use of stents and the length of time for stenting. Scintigraphy should be able to provide quantitative data even when stents are in place. Thus, scans might be helpful with the decision to change or remove a stent. Scintigraphy could also be used to indicate when an indwelling endoprotheses is becoming occluded with biliary "sludge" and requires changing. This technique may also prove useful in the assessment of the results of balloon dilatation, and in the following-up of patients with sclerosing cholangitis.

In summary, iminodiacetic acid scintigraphy appears to be an excellent method for following patients with biliary enteric anastomoses. More data need to be obtained, however, on "normal" hepatic uptake and clearance rates and times, as well as on intestinal uptake time. Baseline scans should probably be performed approximately three months after surgery. In patients with long-term stents, follow-up scans can be done prior to and at three months after stent removal. In addition to serial liver function tests, scintigraphy should probably be performed on at least a yearly basis and whenever symptons or liver function tests suggest a problem. Even in the absence of symptoms, a significant change in function as determined by scintigraphy may warrant further workup including transhepatic cholangiography. With this algorithm, the hope is that anastomotic strictures will be detected and treated earlier and that further liver damage will be minimized.

Henry A. Pitt
Associate Professor and Vice Chairman
Department of Surgery
Johns Hopkins Medical Institutions
Baltimore, Maryland, USA
